# Insight into the structure and molecular mode of action of plant paired NLR immune receptors

**DOI:** 10.1042/EBC20210079

**Published:** 2022-09-30

**Authors:** Yuxuan Xi, Stella Cesari, Thomas Kroj

**Affiliations:** PHIM Plant Health Institute, Univ Montpellier, INRAE, CIRAD, Institut Agro, IRD, Montpellier, France

**Keywords:** immune receptor, phytopathology, plant

## Abstract

The specific recognition of pathogen effectors by intracellular nucleotide-binding domain and leucine-rich repeat receptors (NLRs) is an important component of plant immunity. NLRs have a conserved modular architecture and can be subdivided according to their signaling domain that is mostly a coiled-coil (CC) or a Toll/Interleukin1 receptor (TIR) domain into CNLs and TNLs. Single NLR proteins are often sufficient for both effector recognition and immune activation. However, sometimes, they act in pairs, where two different NLRs are required for disease resistance. Functional studies have revealed that in these cases one NLR of the pair acts as a sensor (sNLR) and one as a helper (hNLR). The genes corresponding to such resistance protein pairs with one-to-one functional co-dependence are clustered, generally with a head-to-head orientation and shared promoter sequences. sNLRs in such functional NLR pairs have additional, non-canonical and highly diverse domains integrated in their conserved modular architecture, which are thought to act as decoys to trap effectors. Recent structure–function studies on the *Arabidopsis thaliana* TNL pair RRS1/RPS4 and on the rice CNL pairs RGA4/RGA5 and Pik-1/Pik-2 are unraveling how such protein pairs function together. Focusing on these model NLR pairs and other recent examples, this review highlights the distinctive features of NLR pairs and their various fascinating mode of action in pathogen effector perception. We also discuss how these findings on NLR pairs pave the way toward improved plant disease resistance.

## Introduction

Plants have an efficient immune system that is activated by the detection of microorganisms through receptors located on the surface or inside their cells [1]. The main class of intracellular immune receptors in plants are nucleotide-binding and leucine-rich repeat domain proteins (NLRs). They act by recognizing inside the host cell effector proteins secreted by pathogens during infection. This recognition is either direct, by formation of an effector/NLR complex that eventually contains additional plant proteins, or indirect, involving the detection of host protein modifications caused by effectors [2,3]. NLR-mediated effector recognition triggers strong immune responses in the infected cell, as well as in surrounding cells and systemic tissue, which involves complex and largely unexplored signal transduction networks [4]. Due to the strong immunity conferred by NLRs, they are essential for the protection of crops from pathogens and pests and corresponding genes are extensively used as disease resistance (*R*) genes for the breeding of disease resistant varieties [2,5].

NLRs are members of the Signal-Transduction ATPases with Numerous Domain (STAND) superfamily that act as signaling hub proteins in all kingdoms of life [6,7]. STAND proteins are characterized by a central signaling and multimerization ATPase domain named NOD (nucleotide binding and oligomerization domain), which controls the activity of the protein by its nucleotide-binding status. Binding of ADP stabilizes the inactive, closed conformation of the STAND proteins [8,9]. Signal recognition leads to conformational changes allowing release of ADP and binding of ATP which stabilizes the active, open conformation of the protein that aggregates to supramolecular homomeric complexes or heteromeric complexes composed of different STAND proteins [10–13]. In addition to the central highly conserved NOD domain, STAND proteins contain a signal recognition domain formed by a C-terminal repeat domain (e.g. ankyrin, armadillo repeat or leucine-rich repeat domain) and an N-terminal signaling domain that becomes operational through proximity-induced activation [6,7].

The central NOD domain of plant NLRs belongs to the NB-ARC subfamily (nucleotide-binding adaptor shared by the human apoptotic regulator APAF-1, plant resistance (R) proteins and Caenorhabditis elegans CED-4) and is generally combined with a C-terminal leucine-rich repeat (LRR) domain and an N-terminal signaling domain that is predominantly a coiled-coil (CC), a resistance to powdery mildew 8 (RPW8) or a Toll/IL1 receptor/resistance proteins (TIR) domain [6,7]. CC-NLRs (CNLs), RPL8-NLRs (RNLs) and TIR-NLRs (TNLs) evolved independently and therefore form distinct classes that are well-differentiated by phylogenetic analysis of the NOD domain [14]. Recent work revealed that the activated TIR domain acts as a nicotinamide adenine dinucleotide (NAD^+^) hydrolase (NADase), producing potentially a low molecular weight signaling molecule [15,16]. The CC domain of ZAR1 was found to form an ion channel upon multimerization [17]. In addition to the three canonical domains, some NLRs contain additional, generally non-canonical integrated domains (IDs) that are mostly located at the C-terminus but may also be inserted between the N-terminal domain and the NOD or at the N-terminus [3,18–20]. These IDs are extremely diverse and many resemble known effector target proteins, regulators of immune signaling or co-factors of NLRs. Therefore, they were proposed to act as decoys that sense effectors in a direct manner by physical binding or indirectly by perceiving their activity [18,21].

While most NLRs act as singletons, *R* gene cloning identified cases where pairs of physically linked NLRs composed of either two TNLs or two CNLs, confer immunity to pathogens (Table 1) [22,23]. Genetic clustering and one-to-one functional co-dependence distinguish NLR pairs from other cases where NLRs interact cooperatively. Prominent examples for these alternative forms of cooperativity between NLRs are the RNLs ADR1, NRG1 and their paralogs, that act downstream of TNLs in dicot plants and the NRC CNLs that are required for the activity of a specific CNL clade expanded in the genomes of *Solanaceous* plants [24–29].

**Table 1:** *R* genes encoding NLR pairs

NLR pair	Type	Orientation	Host plant	Pathogen recognized (AVR)	Integrated domain (ID)	ID Location	Reference
RGA4/RGA5	CNL/CNL-ID	Head-to-head	Rice	*M. oryzae* (AVR-Pia/AVR1- CO39)	HMA	C-terminus of RGA5	[38]
Pik-1/Pik-2 alleles	CNL-ID/CNL	Head-to-head	Rice	*M. oryzae* (AVR-Pik specific alleles)	HMA	Between the CC and NB of Pik-1	[37]^*^
Pi5-1/Pi5-2 Pii-1/Pii-2	CNL/CNL-ID	Head-to-head	Rice	*M. oryzae* (AVR-Pii)	NOI (Nitrate-Induced)	C-terminus of Pi5-2 and Pii-2	[33,34]
RRS1/RPS4	TNL-ID/TNL	Head-to-head	Arabidopsis	*Pseudomonas syringae*(AvrRps4) *Ralstonia solanacearum* (PopP2), *Colletotrichum higginsianum*	WRKY	C-terminus of RRS1	[32,36]
RRS1B/RPS4B	TNL-ID/TNL	Head-to-head	Arabidopsis	*Pseudomonas syringae* (AvrRps4)	WRKY	C-terminus of RRS1B	[65]
RPP2A/RPP2B	TNL-ID/TNL	Head-to-tail	Arabidopsis	*Hyaloperonospora arabidopsidis* (Cala2 isolate)	TIR:NB-DUF640/CC	N-terminus /Between the second NB and LRR of RPP2A	[86]
Lr10/RGA2	CNL/CNL-ID	Head-to-head	Wheat	*Puccinia triticina*	NB (Nucleotide-Binding)	Between the CC and NB of RGA2	[87]
Fom-1/Prv	TNL/TNL-ID	Head-to-head	Melon	*Fusarium oxysporum f.sp. melonis* (races 0 and 1) *Papaya ring-spot virus*	NB	C-terminus of Prv	[88]
Pias1/Pias2	CNL/CNL-ID	Head-to-head	Rice	*M. oryzae* (AVR-Pias)	DUF761 (Domain of Unknown Function 761)	C-terminus of Pias2	[89]
RGA1/Rpg5	CNL/CNL-ID	Head-to-head	Barley	*Puccinia graminis*	STPK (serine/threonine protein kinase)	C-terminus of Rpg5	[90]

* The first Pik pair Pikm-1/Pikm-2 was reported in this publication. Other cloned Pik pairs are listed in Figure 3.

In this review, we highlight the distinctive features of NLR pairs and their diversity and provide an overview of the different molecular mode of action of paired NLR immune receptors that confer disease resistance to plants. We also discuss how recent exciting findings on NLR pairs pave the way toward improved plant disease resistance.

## Distinctive features of NLR pairs enable their detection in plant genomes

A distinctive feature of paired *NLR R* genes is their genetic clustering. They are predominantly arranged in a head-to-head orientation and share, in the case of functionally validated pairs, an intergenic region of several hundred to several thousand nucleotides. An additional shared characteristic is the integration of an additional mostly non-canonical domain into one NLR of the pair [18]. NLR pairs can be formed either by TNLs or by CNLs, and representatives from multiple clades of these NLR classes are found within pairs, which indicates that NLR pairing occurred multiple independent times during plant evolution [30–32]. In most cases, the two *NLRs* of a pair are from two different clades that are generally separated by clades of singleton NLRs. This situation suggests that such NLR pairs emerged by assembly of pre-existing NLRs into a functional unit [30–32]. However, other NLR pairs originated potentially from a recent duplication of one NLR and integration of an ID into one of the copies. An *NLR* pair that originated probably by this mechanism is *Pi5-1*/*Pi5-2* (allelic to *Pii-1/Pii2*) from the rice blast resistance locus *Pi5*/*Pii*, that share high sequence similarity [33–35].

Based on the distinctive features of paired *NLR R* genes (i.e. clustering of divergent NLRs, head-to-head orientation and presence of an ID in one of them), genomic analysis identified numerous and diverse paired NLRs in plant genomes. For instance, a comparative genomics analysis of 13 rice species identified 165 NLR pairs with a head-to-head orientation among 5408 NLRs, 1/3 of which included an NLR-ID [31]. Another analysis of approximately 6200 NLRs in nine cereal genomes identified 450 NLR-IDs, 50 of which showed the features of paired NLRs. Interestingly, IDs were found at low frequency in many NLR clades but at high frequency in only three clades [30]. Among them, the Major Integrator Clade 1 (MIC1) stood out as it contains almost 30% of all NLR-IDs and is exclusively paired with NLRs from one specific other clade [30].

## Three model pairs for the understanding of the molecular mechanism of paired NLRs

**Figure 1:**
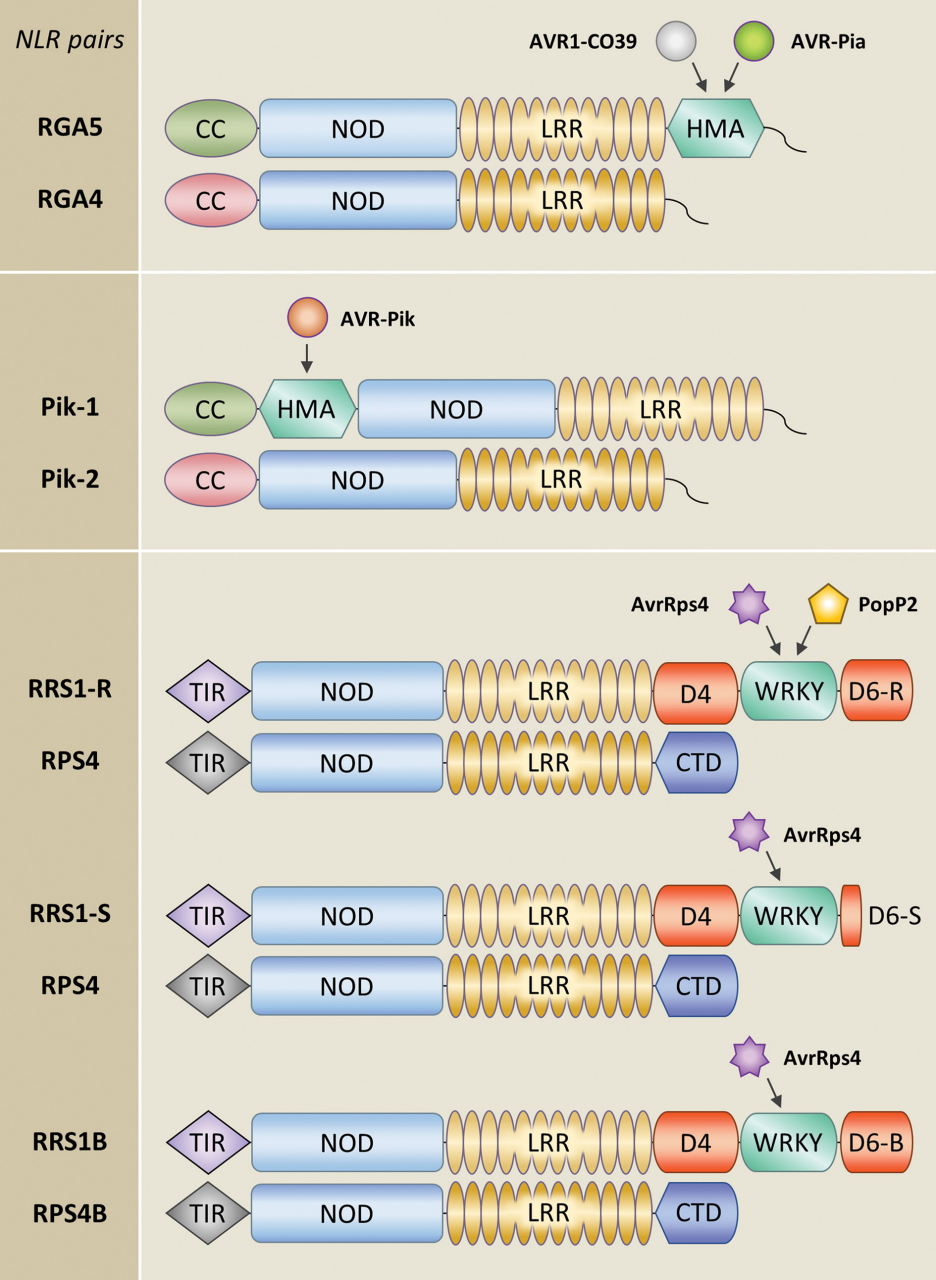
Models of plant paired NLRs and the pathogen effectors they specifically recognize RGA5 and Pik-1 act as sNLRs in the rice CNL pairs RGA4/RGA5 and Pik-1/Pik-2. Their HMA ID located respectively after the LRR or between the NOD and LRR domains binds MAX effectors from the blast fungus *P. oryzae*. This binding is crucial for effector recognition. RRS1 is the sNLR in the TNL pair RRS1/RPS4 from *A. thaliana*. Its WRKY ID is essential for the detection of the bacterial effectors PopP2 and AvrRps4. The C-terminal domain (CTD) of RPS4 is composed of a post-LRR (PL) domain present at this position in most TNLs followed by sequences without similarity to other proteins [85]. The PL domain adopts a β-jelly-roll and immunoglobulin-like β-sandwich fold as revealed by the RPP1 and ROQ1 structures [78,79], where this domain acts together with the LRR in effector binding. The Domain 4 (D4) of RRS1, located between the LRR and the WRKY domain, also contains a PL domain that is however degenerated [85]. Domain 6 (D6) located at the C-terminus of RRS1, downstream of the ID, has no homology to other proteins. A length polymorphism between the S and R alleles of RRS1 in D6 determines the recognition spectrum. RRS1-R characterized by an extended D6 of ∼100 amino acids recognizes both bacterial effectors, while RRS1-S that has a short D6 of ∼20 amino acids only detects AvrRps4 but not PopP2. The paralogous pair RRS1B/RPS4B, which has roughly 70% amino acid sequence identity with RRS1/RPS4, recognizes only AvrRps4 despite its long D6 that is largely similar to the one of RRS1-R.

**Figure 2:**
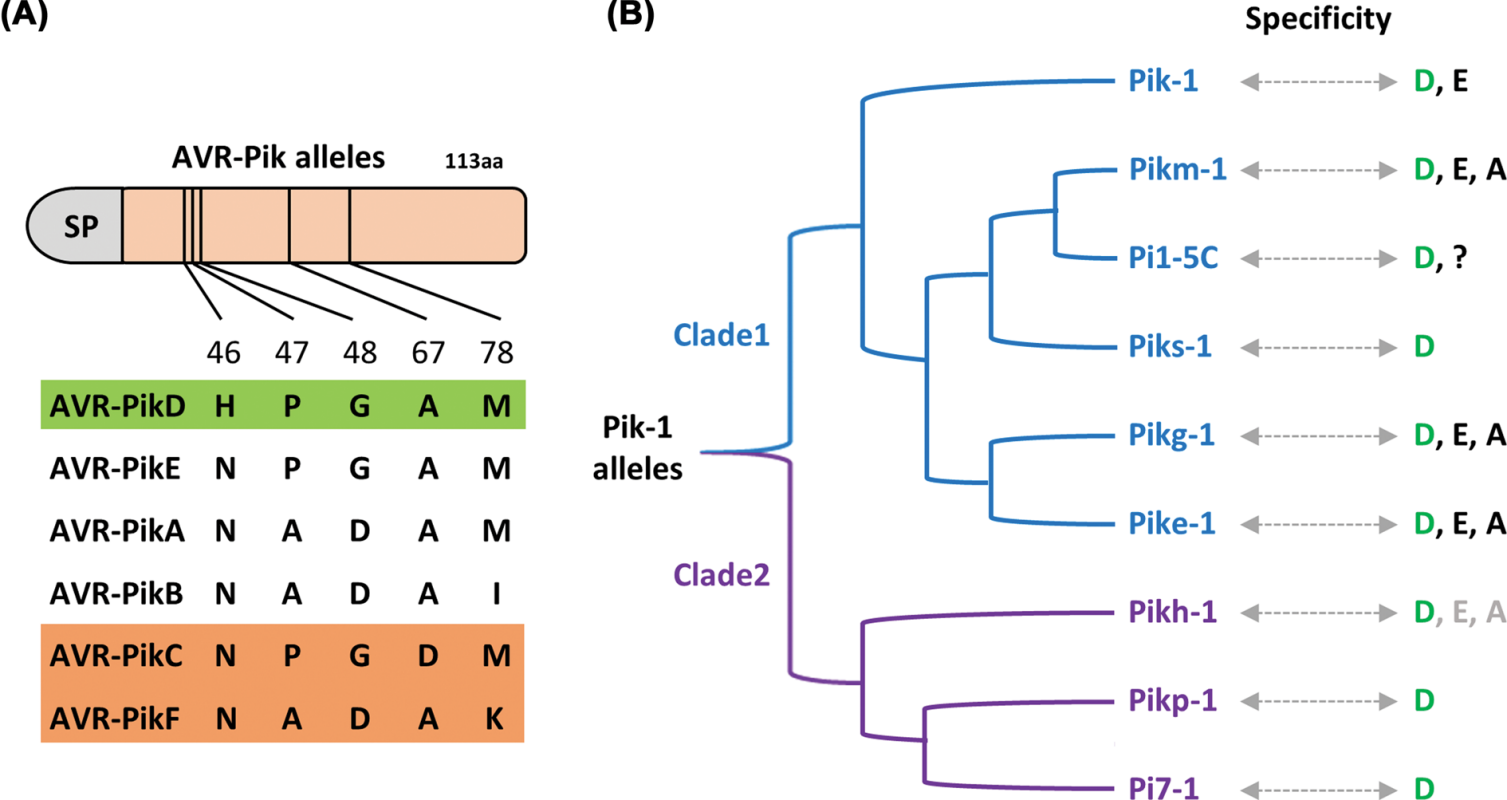
Diversity and recognition specificity between alleles of Pik-1 and AVR-Pik (**A**) AVR-Pik alleles differ at 5 polymorphic sites located in the N-terminal extension of the MAX effector structure. AVR-PikC and AVR-PikF (orange filled rectangle) escape detection by all known Pik-1 alleles. SP stands for signal peptide. (**B**) Various Pik-1 alleles from two major clades recognize AVR-PikD (green filled rectangle in panel A) but differ in their ability to detect the alleles A and E. The recognition specificity of Pi1-5C is not well defined as only AVR-PikD has been tested.

Knowledge on the molecular mode of action of paired NLRs is still limited and stems mainly from studies on three model pairs (Figure 1): the TNL pair RRS1/RPS4 from *Arabidopsis thaliana* that confers resistance to bacterial and fungal pathogens [32,36], and the two unrelated CNL pairs RGA4/RGA5 and Pik-1/Pik-2 from rice. Each of these rice CNL pairs confers resistance to the fungus *Pyricularia oryzae* (syn. *Magnaporthe oryzae*), which causes the devastating blast disease on cereals [37,38]. The RRS1/RPS4 pair recognizes AvrRps4 from *Pseudomonas syringae* and the bacterial acetyltransferase PopP2 from *Ralstonia solanacearum*, both of which are effectors secreted into host cells by the bacterial type three secretion system [39,40]. The RGA4/RGA5 pair detects the *P. oryzae* effectors AVR-Pia and AVR1-CO39 [38,41], while Pik-1/Pik-2 recognizes the effector AVR-Pik [37,42]. Pik-1/Pik-2 mediated recognition of AVR-Pik is allele specific since there are at least 6 AVR-Pik alleles (A to F), varying at 5 polymorphic sites in *P. oryzae* populations and various different functional alleles of Pik-1/Pik-2, with distinct but overlapping recognition specificities (Figure 2) [37,43–50]. The three *P. oryzae* effectors recognized by RGA4/RGA5 or Pik-1/Pik-2 have no apparent sequence identity. However, they possess the same three-dimensional structure, comprising a 6-stranded beta sandwich stabilized by disulfide bonds, which is characteristic of the *Magnaporthe* AVRs and ToxB-like (MAX) effector family that is widespread in ascomycete fungal pathogens and strongly expanded in *P. oryzae* [51].

## Commonalities and specificities in the mechanisms of effector detection by paired NLRs

Detailed molecular studies on the mechanism of effector recognition by these model NLR pairs have identified important commonalities. In all cases, effector recognition mainly involves one NLR of the pair that is therefore named sensor NLR (sNLR), while the other NLR acts in the activation of downstream immune signalling and is therefore named the helper NLR (hNLR) [41,43,52–55]. The sNLR always carries a non-canonical ID, which, in all cases, plays a crucial role in the process of effector detection.

**Figure 3:**
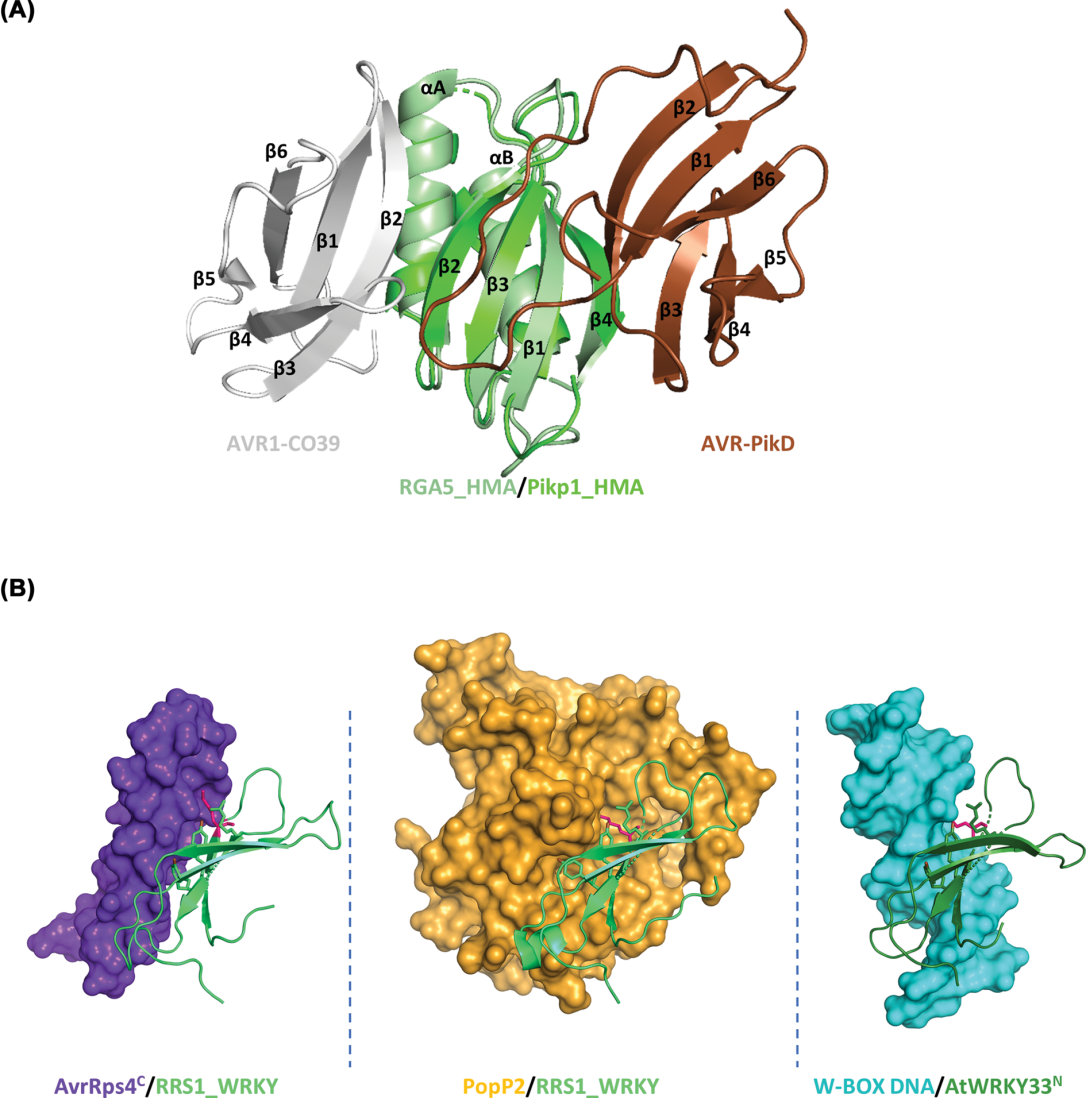
The three-dimensional models of ID/effector complexes (**A**) An overlay of the structures of the AVR1-CO39/RGA5_HMA complex (gray/pale green, PDB: 5ZNG) and the Pikp-1_HMA/AVR-PikD complex (green/brown, PDB: 5A6W) shows that AVR1-CO39 and AVR-PikD bind the HMA domains through opposite surfaces. (**B**) Models of AvrRps4C/RRS1_WRKY (purple/limegreen, PDB: 7P8K), PopP2/RRS1_WRKY (orange/limegreen, PDB: 5W3X) and W-box DNA /AtWRKY33N (cyan/forest green, PDB: 6J4G) show that the bacterial effectors bind the DNA-binding surface of the WRKY-domain (adapted from Mukhi et al., 2021). The side chains of some key residues mediating the interaction in the complexes are shown in the models, in particular a conserved Lysine from the WRKY consensus sequence that protrudes into a surface cleft of the contacted molecules is colored in hotpink.

RGA5 and Pik-1 are the sNLRs within the two rice CNL pairs RGA4/RGA5 and Pik-1/Pik-2, and both of them carry a heavy metal associated (HMA) ID, which is located downstream of the LRR domain in RGA5 and inserted between the CC and the NOD in Pik-1 (Figure 1) [41]. HMA domains have a conserved α/β sandwich structure that binds heavy metals through a conserved CxxC motif and occur in heavy metal transporters and chaperons. The HMA IDs are paralogous to an expanded family of HMA plant proteins (HPPs) and HMA isoprenylated plant proteins (HIPPs), collectively referred to as sHMAs (small HMAs), whose function is largely unknown and whose metal binding motifs are often degenerated [56,57]. In both RGA5 and Pik-1, the effectors bind physically to the HMA domain, and this binding is crucial for recognition and activation of immune responses [41,43,53]. However, structure–function analysis revealed that the underlying binding mechanisms and the binding interfaces are completely different in both systems [53,58]. Indeed, the binding interface in the AVR1-CO39/RGA5_HMA complex is formed by the β1 and β2 stands of the MAX effector and the αA helix and the β2 strand of the HMA (Figure 3) [42,53,58]. The structure of the AVR-Pia/RGA5_HMA complex has not been determined but NMR titration, functional analyses and 3D modelling indicate that AVR-Pia binds RGA5_HMA in a highly similar manner as AVR1-CO39 [59,60]. In contrast to this, in the AVR-Pik/Pik-1_HMA complex, binding is conferred by the β2 and β3 strands of AVR-Pik as well as its long N-terminal extension, and by three main interaction areas located on the β-sheet of the Pik-1_HMA. Among them, interface 1 is formed by residues in β1, interface 2 involves residues from β2 and β3 and interface 3 consists of β4 and proximal residues from the following loop (Figure 3). These fundamental differences in the ID/effector complexes indicate that HMA-mediated effector recognition has evolved completely independently in RGA5 and Pik-1.

The marked allele specificity in the AVR-Pik Pik-1/Pik-2 interaction is a characteristic of this NLR pair and recent studies deciphered its mechanistic basis and its evolutionary origin. They showed that key effector-binding residues in interfaces 2 and 3 are polymorphic between Pikp-1 and Pikm-1, which results in drastically different contributions of these two surfaces to AVR-Pik-binding in the two cases and in different recognition specificities [42]. Phylogenetic analysis and ancestral sequence reconstitution indicate that Pikm-1 and Pikp-1 define two distinct Pik-1 clades that evolved independently from a common ancestor, which was unable to sense AVR-Pik (Figure 2) [61]. Evolution toward binding and recognition of AVR-Pik took two different mutational paths leading in the Pikp-1 clade to crucial changes in binding interface 2 and in the Pikm-1 clade to critical mutations in interface 3. This provides a unique example of how effector recognition emerged through evolution in plant immune receptors.

**Figure 4:**
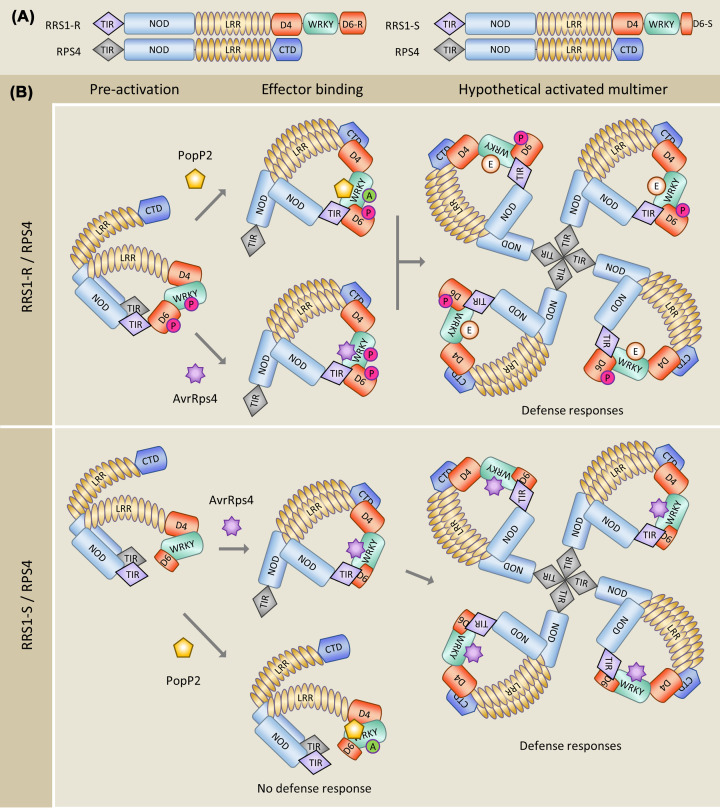
Models for the specific recognition of PopP2 and AvrRps4 by RRS1 and RPS4 (**A**) Structures of RPS4, RRS1-R and RRS1-S. (**B**) Regulation of the RRS1/RPS4 receptor complex relies on complex inter and intramolecular interactions. Key features of the pre-activation state, in which RPS4 is repressed by RRS1, are (1) interactions between the RRS1 domain 4 (D4) and its WRKY domain (D5) that trap RRS1 in a closed conformation, as well as (2) interactions between the TIR domains of RRS1 and RPS4 that prevent activation of signalling by the RPS4 TIR domain [77,80]. In RRS1-R, the long C-terminal domain 6 (D6) extends to the RRS1-R TIR domain even in the pre-activation state, whereas in the RRS1-S/RPS4 pre-activation state, the TIR domain and the C-terminus (D5D6) are not in close proximity. In both pairs, AvrRps4 promotes interactions between the C terminus (D5D6) and the TIR domain of RRS1, which de-represses the hetero-complex. PopP2 acetylates the WRKY domain (D5) of both RRS1-R and RRS1-S, but is only perceived by RRS1-R through intramolecular TIR/D5D6-R interaction [81]. Effector-triggered TIR/D5D6 interactions in both RRS1-R and -S relieve the inhibitory interaction between the TIR domains of RRS1 and RPS4, allowing self-association of RPS4 TIR domains. In addition, interactions between RRS1 D4 and the RPS4 C-terminal domain (CTD) contribute to the activation of the receptor complex [80]. All these processes are believed to culminate in the formation of a resistosome complex that triggers immune signalling probably by proximity-induced activation of the intrinsic NADase activity of RPS4 TIR, a mechanism illustrated by the crystal structures of the TNLs ROQ1 and RPP1 [78–81]. Recent work showed that the phosphorylation of specific threonine and serine residues in the C-terminus of RRS1-R plays an important role in its regulation and PopP2 responsiveness [81]. The phosphorylation of one specific threonine residue of the WRKY domain is required to maintain RRS1-R in its inactive state. Phosphorylation at other sites located in D6 strengthens the interaction of the C terminus and the TIR domain of RRS1 and is required for activation by PopP2 but not for AvrRps4 responsiveness. P: phosphorylation, A: acetylation, E: effector-triggered modification or binding.

Additional mechanisms and even higher versatility in effector recognition were revealed in the RRS1/RPS4 pair. Effectors are sensed by the sNLR RRS1 that carries at its C-terminus an integrated WRKY domain, a DNA binding domain that characterizes an extended transcription factor family in higher plants and that has a crucial role in the regulation of biotic stress responses and immunity [54,55,62]. AvrRps4 binds with high affinity to the RRS1 WRKY domain and a crystal structure of this complex shows that the binding interface overlaps with the DNA-binding surface of the WRKY domain [63] (Figure 3). PopP2 also interacts with the WRKY domain of RRS1 through an overlapping but much bigger surface than that involved in AvrRps4 binding, and acetylates proximal lysine residues, in particular specific lysines in the WRKY consensus motif that are crucial for DNA binding [64]. There are two alleles of RRS1 that differ in the length of their C-terminal domain 6 (D6) located after the WRKY domain (Figure 1). RRS1-R that recognizes both AvrRps4 and PopP2 has an extended D6 while RRS1-S that detects only AvrRps4 has a short D6 due to a premature stop-codon [32,36] (Figure 4). Certain *A. thaliana* accessions possess in addition the paralogous RRS1B/RPS4B pair that only recognizes AvrRps4. RRS1B has 70% overall sequence identity with RRS1, carries a significantly different WRKY and a long D6. Its WRKY domain is bound and acetylated by PopP2, as in the case of RRS1-S, indicating that acetylation activity is not sufficient for PopP2 recognition [65].

Another effector detection mechanism is involved in the specific recognition of the *P. oryzae* effector AVR-Pii by the rice CNL pair Pii-1/Pii-2, which is allelic and functionally equivalent to Pi5-1/Pi5-2 [34]. Effector recognition by Pii-1/Pii-2 requires the rice protein Exo70-F3, a component of the exocyst complex mediating polarized exocytosis, which binds AVR-Pii [66]. Pre-published data suggest that Pii-2 interacts with Exo70-F3 through its nitrate-induced (NOI) ID and thereby relies on this guardee or decoy protein to monitor the presence of AVR-Pii in an indirect manner [67].

Taken together, our current knowledge shows that although specific recognition of effectors by paired NLRs always relies on the sNLR and involves its ID, different molecular mechanisms are mobilized among the different pairs for this recognition. This complexity mirrors the diversity of effector perception mechanisms observed in NLRs acting as singletons.

## Effector recognition by NLR pairs supports an integrated decoy model

The prominent role of IDs in effector recognition by sNLRs and their similarity to plant proteins targeted by pathogen effectors suggest that they are mimics of true effector targets [18]. Strongest support for this integrated decoy model comes from investigation of the PopP2/RRS1-R/RPS4 system, where it has been demonstrated that WRKY transcription factors are virulence targets of the recognized effector and that the interaction mechanisms with the ID and the targets are similar [54,55]. Indeed, PopP2 acetylates the RRS1 WRKY domain in the same manner as the DNA-binding site of various WRKY transcription factors, some of which are well-documented immune regulators (Figure 3). This abolishes their DNA binding activity and the regulation of target genes resulting in attenuated immune responses and increased bacterial virulence. AvrRps4 also interacts with genuine WRKY transcription factors, which inhibits their binding to target promoter elements [63].

The fact that the rice HPP Pi21 is a well-documented blast susceptibility factor [68] supports the hypothesis that sHMA proteins may be targets of *P. oryzae* effectors and in particular those of the MAX family. In addition, pre-published data further support this hypothesis since they show that AVR-Pik binds various rice HPPs and HIPPs, which results in their stabilization, and that one of them acts as a susceptibility factors to rice blast [69]. Crystal structure analysis revealed that binding of AVR-Pik to sHMA proteins is highly similar to Pik-1_HMA binding, providing additional mechanistic support for the integrated decoy model [70].

## Translation of effector detection into immune activation

How effector recognition by sNLRs is translated into immune activation and how paired NLRs interact is largely unexplored but the already existing knowledge indicates important mechanistic diversity. In RGA4/RGA5 and RRS1/RPS4, the hNLR is autoactive and repressed at the resting state by the sNLR [52,71–73]. This repression is relieved by effector recognition. Consistent with this, overexpression of the hNLR results in cell death and mutation of its p-loop (a motif in the NOD required for ATP binding) but not that of the sNLR, inactivates the NLR pair. Interestingly, the ID of RGA5 is dispensable for hNLR repression while the WRKY domain of RRS1 is strictly required and multiple non-repressive RRS1 mutants with point mutations in the WRKY domain were identified. Deletion mutants in other parts of both sNLRs invariably loose hNLR repressor activity. Unlike this, Pik-1/Pik-2 show cooperative interaction [74]. Neither of the NLRs in the pair can activate cell death on its own and P-loop mutations in either of them abolish the pair’s activity. Interestingly, mispaired Pik-1/Pik-2 alleles become either autoactive, or have attenuated activity, and a specific residue in the NB-domain mediating proper pairing between Pikm and Pikp alleles has been identified [75].

**Figure 5:**
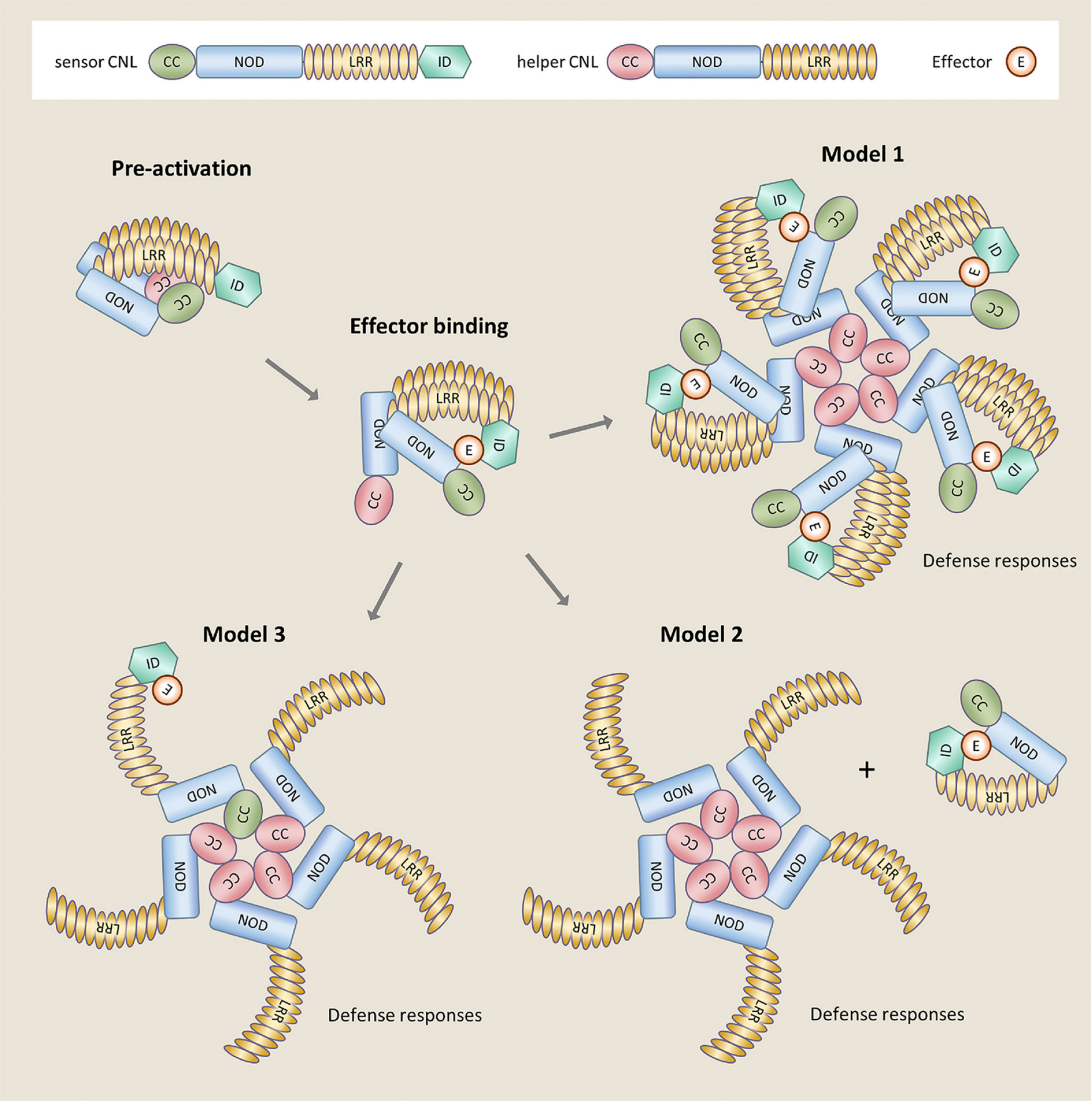
Hypothetical models for effector recognition by CNL pairs The upper box shows the secondary structure of a sensor CNL, with the ID at the C-terminus and a helper CNL, as well as the recognized effector. In the pre-activation state the sCNL and hCNL form an inactive complex with CC hetero-interactions. In certain pairs, such as RGA4/RGA5, the sNLR maintains the autoactive hNLR in a repressed state in this pre-activation complex. Binding of the effector to the sCNL is thought to trigger molecular rearrangements that activate the hetero-complex and induce the formation of a ZAR1-like resistosome where the hCNL CC domains self-associate. Model 1 assumes that the effector-bound sCNL is functionally analogous to the PBL2/RKS1 complex in the ZAR1 resistosome. In this model, the sCNL/effector complex associates to the periphery of the resistosome that is formed by the association of the hCNL into a pentameric wheel-like multimer. Model 2 implies that the sCNL is released from the pre-activation complex upon effector binding and that the resistosome is only composed of the hCNL. Finally, the sNLR could be part of the inner pentameric ring of the resistosome together with the hCNL as depicted in Model 3. In the case of ZAR1, the self-association of the CC domain in the resistosome creates a cation channel that inserts into in the plasma membrane and triggers calcium influx, immunity and cell-death. Such activity has not been reported for CNL pairs but sequence similarity between the CC domains of ZAR1 and hCLRs suggests that they could act by similar mechanisms.

In all studied paired NLRs, sNLRs and hNLRs form heteromeric complexes in the resting and in the activated state, as mainly shown by co-immunoprecipitation experiments [52,74,76,77]. It is therefore tempting to speculate that they form heterodimers when inactive and aggregate upon effector perception into the higher order ring-like complexes characteristic for STANDs, which are called resistosomes for plant NLRs [10–12,78,79] (Figures 4 and 5). However, the molecular details of sNLR/hNLR complex formation and the mechanism of activation of immune signaling by NLR pairs upon effector recognition is poorly defined and the stoichiometry and composition of these complexes are unknown. For RGA4/RGA5 and Pik-1/Pik-2, it is also unclear which domains mediate these interactions and which intra and intermolecular interactions change upon activation (Figure 5). For RRS1/RPS4, multiple inter and intramolecular interactions have been identified in the inactive and active receptor complex and mechanistic models for its regulation and reconfiguration upon effector-mediated de-repression were established [77,80,81] (Figure 4).

## Applications and perspectives for paired NLR research

The detailed insight into the recognition specificity of the described model NLR pairs recently enabled important progress in their engineering for the detection of novel effectors. Structure-guided modifications in the effector-binding interface of the HMA domain of Pikp-1 enabled perception of multiple AVR-Pik alleles and thereby broadened recognition specificity of the mutant receptor to that of Pikm-1 [82]. RGA5 mutants where the AVR1-CO39-binding surface in the HMA was engineered to interact with AvrPib, another *P. oryzae* MAX effector, recognized this new effector and conferred together with RGA4 immunity in rice [83]. Introduction of the AVR-PikD binding residues of the Pikp-1 HMA domain into the HMA domain of RGA5 created a high-affinity binding surface for this effector and RGA5 variants carrying this engineered binding surface perceived AVR-PikD as well as its original effectors in the model plant *N. benthamiana* [60]. However, they did not confer extended disease resistance specificity against *P. oryzae* in transgenic rice plants, revealing important knowledge gaps. Next steps are more far-reaching modifications of the ID or their exchange by completely new effector binding domains. In this sense, pre-published data reporting that the replacement of the HMA in Pikm-1 by a GFP-binding nano-body allows GFP detection in the *N. benthamiana* model plant provides very exciting perspectives [84].

However, despite the wealth of information on the major paired NLR models, many fundamental questions remain largely unexplored such as the specific roles of the NOD and LRR domains and the mechanisms of complex formation in the resting and the active state. Deciphering the composition and stoichiometry of such complexes and structure information on full-length proteins will be particularly critical to progress in this research area. In addition, it will be important to extend functional investigation to additional models to explore the diversity of mechanisms employed for effector recognition and immune activation by NLR pairs.

## Summary

- NLR pairs are coded by two genetically clustered and coevolved *NLR* genes with a characteristic head-to-head orientation and a one-to-one functional co-dependence.
- They consist of one sNLR carrying an ID and mediating effector detection and one hNLR activating immune signaling.
- Effector recognition relies on the sNLR's ID, which acts by various mechanisms according to an integrated decoy model.
- The functional interaction between the sNLR and the hNLR relies on intermolecular interactions in a heteromeric receptor complex. Effector detection is believed to trigger conformational changes that remodel sNLRs/hNLRs interactions and culminate in the formation of a signaling competent oligomeric resistosome complexes.
- The recent creation of new and defined recognition specificities for paired NLRs using molecular engineering demonstrates the potential of this class of immune receptor for knowledge-driven sustainable crop protection.
- Determining the composition and stoichiometry of sNLR/hNLR complexes at the resting and active state and resolving their three-dimensional structures are major objectives for future research on NLR pairs.

## Abbreviations

CC: coiled-coil
HIPP: HMA isoprenylated plant protein
HMA: heavy metal associated
hNLR: helper NLR
HPP: HMA plant protein
MIC1: Major Integrator Clade 1
NLR: nucleotide-binding domain and leucine-rich repeat protein
sNLR: sensor NLR
TIR: Toll/Interleukin1 receptor
